# A prospective multicentre study evaluating the outcomes of the abdominal wall dehiscence repair using posterior component separation with transversus abdominis muscle release reinforced by a retro-muscular mesh: filling a step

**DOI:** 10.1186/s13017-023-00485-9

**Published:** 2023-03-03

**Authors:** Tamer A. A. M. Habeeb, Abdulzahra Hussain, Vishal Shelat, Massimo Chiaretti, Jose Bueno-Lledó, Alfonso García Fadrique, Abd-Elfattah Kalmoush, Mohamed Elnemr, Khaled Safwat, Ahmed Raafat, Tamer Wasefy, Ibrahim A. Heggy, Gamal Osman, Waleed A. Abdelhady, Walid A. Mawla, Alaa A. Fiad, Mostafa M. Elaidy, Wessam Amr, Mohamed I. Abdelhamid, Ahmed Mahmoud Abdou, Abdelaziz I. A. Ibrahim, Muhammad Ali Baghdadi

**Affiliations:** 1 Department of General Surgery, Faculty of Medicine, Zagazig University, 1 Faculty of Medicine Street, Zagazig, Egypt; 2 Homerton University Hospital, London, UK; 3 Department of General Surgery, Tan Tock Seng Hospital, Singapore, Singapore; 4 Department of General Surgery, Surgical Specialities and Organ Transplantation “Paride Stefanini”, Sapienza University of Rome, Rome, Italy; 5 Unit of Abdominal Wall Surgery, Department of General Surgery, Hospital Universitari i Politècnic la Fe, Valencia, Spain; 6 Department of General Surgery, Instituto Valenciano de Oncologia, Valencia, Spain; 7 General Surgery Department, Al-Azhar University, Cairo, Egypt; 8 Obstetrics and Gynecology Department, Faculty of Medicine, Zagazig University, Zagazig, Egypt

**Keywords:** Abdominal wall dehiscence, Incisional hernia, Posterior component separation, Retro-muscular mesh

## Abstract

**Background:**

This study aimed to evaluate the results of posterior component separation (CS) and transversus abdominis muscle release (TAR) with retro-muscular mesh reinforcement in patients with primary abdominal wall dehiscence (AWD). The secondary aims were to detect the incidence of postoperative surgical site occurrence and risk factors of incisional hernia (IH) development following AWD repair with posterior CS with TAR reinforced by retromuscular mesh.

**Methods:**

Between June 2014 and April 2018, 202 patients with grade IA primary AWD (Björck's first classification) following midline laparotomies were treated using posterior CS with TAR release reinforced by a retro-muscular mesh in a prospective multicenter cohort study.

**Results:**

The mean age was 42 ± 10 years, with female predominance (59.9%). The mean time from index surgery (midline laparotomy) to primary AWD was 7 ± 3 days. The mean vertical length of primary AWD was 16 ± 2 cm. The median time from primary AWD occurrence to posterior CS + TAR surgery was 3 ± 1 days. The mean operative time of posterior CS + TAR was 95 ± 12 min. No recurrent AWD occurred. Surgical site infections (SSI), seroma, hematoma, IH, and infected mesh occurred in 7.9%, 12.4%, 2%, 8.9%, and 3%, respectively. Mortality was reported in 2.5%. Old age, male gender, smoking, albumin level < 3.5 gm%, time from AWD to posterior CS + TAR surgery, SSI, ileus, and infected mesh were significantly higher in IH. IH rate was 0.5% and 8.9% at two and three years, respectively. In multivariate logistic regression analyses, the predictors of IH were time from AWD till posterior CS + TAR surgical intervention, ileus, SSI, and infected mesh.

**Conclusion:**

Posterior CS with TAR reinforced by retro-muscular mesh insertion resulted in no AWD recurrence, low IH rates, and low mortality of 2.5%.

*Trial registration* Clinical trial: NCT05278117.

**Supplementary Information:**

The online version contains supplementary material available at 10.1186/s13017-023-00485-9.

## Introduction

Abdominal wound dehiscence (AWD) is a dreaded complication following a laparotomy. AWD incidence ranges between 2 and 5.5% after elective laparotomy and 8.5–45% after emergency laparotomy and typically occurs between the 6th and 12th postoperative day with up to 25% mortality [1–4]. To avoid evisceration and infection of the abdominal cavity, immediate repair of AWD is recommended [2]. A surgeon has multiple choices to repair an AWD; no one technique is considered “the gold standard”. The options include a conservative approach for small fascial defects [3], negative pressure wound therapy (NPWT) [5–8], mesh reinforcement, component separation (CS) technique [9], and primary repair [10–13]. Despite attention to detail, AWD recurrence occurs in up to 12.9%, and incisional hernia (IH) develops in up to 83% of patients following AWD corrective surgery [10, 14, 15]. Recurrence of AWD increases mortality significantly and increases the risk of IH in survivors. IH is a chronic complication that causes discomfort, pain, and poor quality of life, with a high risk of recurrence after revision surgery [16]. Therefore, reducing IH following AWD repair would significantly impact morbidity, mortality, and quality of life.

In a prior guideline, it was agreed not to create a recommendation on the use of posterior component separation (CS) with transversus abdominis muscle release (TAR) in managing AWD and that its usage should be cautiously and wisely assessed to prevent potential risk for upcoming abdominal wall surgical treatments [17]. However, another study stated that posterior CS combined with TAR was significantly helpful in AWD closure without major complications, but the study's patient population was limited, and the evidence quality was deemed insufficient [9].

This study aimed to evaluate the results of posterior component separation (CS) and transversus abdominis muscle release (TAR) with retro-muscular mesh reinforcement in patients with AWD. The secondary aims were to detect the incidence of postoperative surgical site occurrence (SSO) and risk factors of IH development.

## Methods

### Study design and participants

Between June 2014 and April 2018, 202 patients with complete primary AWD following midline laparotomies were identified at seven hospitals with various surgical departments. The study is solely focused on patients with primary AWD and not IH in the first place. These patients were prospectively identified through the records of emergency laparotomies at respective hospitals and were enrolled; it is a comprehensive sampling including all patients during the study period. Inclusion and exclusion criteria are shown in Table 1. We used Björck's initial classification, published in 2009 and updated in 2016—defined as a clean open abdomen without adherence between the bowel and abdominal wall or fixity [18, 19]. This study was designed following the Declaration of Helsinki guidelines and approved by the Ethics Committee of our universities hospital together with the Strengthening the Reporting of Observational Studies in Epidemiology (STROBE) guidelines.

**Table 1:** Inclusion and exclusion criteria

Inclusion criteria	Exclusion criteria
1. Age ≥ 18 years 2. Previous midline laparotomy 3. No abdominal contamination 4. Grade IA according to Björck's initial classification	1. Grade 1B, 2, 3 and 4 according to Björck's initial classification 2. Primary laparotomy performed through a non-midline incision 3. Age < 18 years 4. Open abdomen 5. If another laparotomy had been performed between the surgery for BA and the end of the follow-up period 6. Concomitant intra-abdominal surgery 7. Abdominal complications during BA 8. Adherent bowel to the defect edge that cannot be separated 9. Presence of intra-abdominal contamination that cannot be drained radiologically 10. History of previous abdominal wound dehiscence repair 11. Stoma exteriorization from the midline primary wound 12. Temporarily wound closure techniques 13. Prior abdominal surgeries other than operation resulted in BA 14. Prior abdominal wall hernia repair with or without mesh 15. Known history of collagen tissue diseases or other related pathologies 16. Previous incisional hernia 17. Patients lost during follow up

Primary AWD is defined as partial or complete separation of the previously approximated wound edges with evisceration that occurs a few days after laparotomy (Additional file 1: Fig. S1). AWD recurrence is the disruption of all abdominal wall layers after the previous repair for primary AWD. For this study, we adopted the Centre for Disease Control (CDC) classification system of SSI as superficial, deep, and organ/space [20]. Surgical site occurrence (SSO) includes surgical site infection (SSI), surgical site hematoma, and surgical site seroma.

We adopted the European Hernia Society (EHS) definition of IH as "any abdominal wall gap with or without a bulge in the area of a surgical scar" [21]. The Clavien-Dindo classification evaluated postoperative morbidity [22]. Increases in peak airway pressures > 12 mmHg or changes in plateau airway pressures > 6 cm H2O over baseline indicate that the fascial closure is under excessive tension, putting patients at risk for respiratory problems and repair failure with recurrence [23].

### Study outcomes and endpoints

The primary outcome was to evaluate the results of posterior component separation (CS) and transversus abdominis muscle release (TAR) with retro-muscular mesh reinforcement in patients with primary AWD. The secondary outcomes were to detect the incidence of postoperative surgical site occurrence (SSO) and risk factors of IH development.

### Perioperative technique

**Fig. 1:**
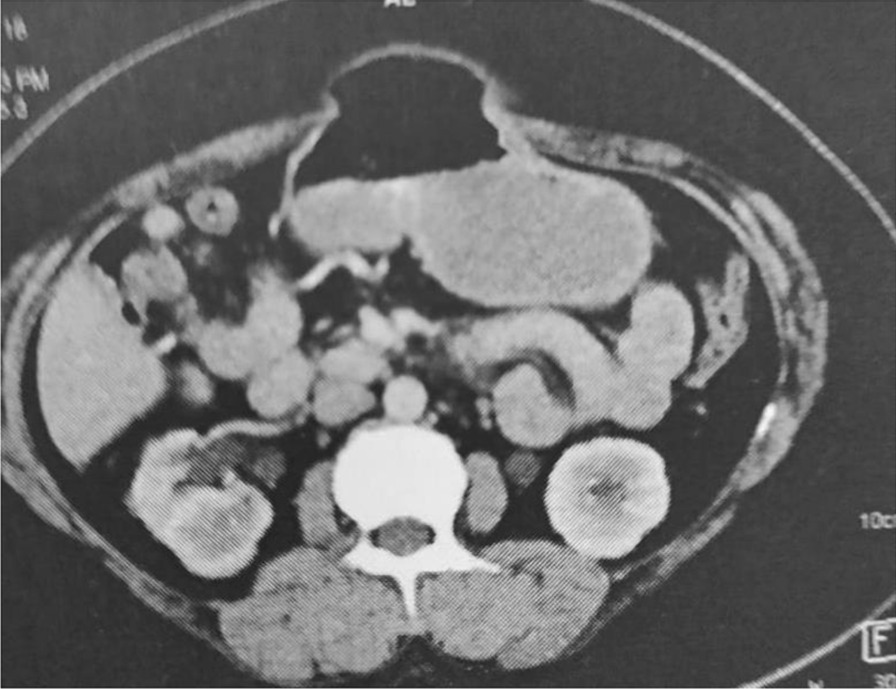
CT of abdomen shows burst abdomen with absence of intra-abdominal abscess

In all patients, abdominal radiographs and ultrasonography (USS) were performed. An abdominal computed tomography (CT) scan was also performed to rule out intra-abdominal abscesses (Fig. 1). Two grams of cefuroxime were administered during the induction of anesthesia, and low molecular weight heparin 4000 IU was used as prophylaxis against deep vein thrombosis.

All procedures were done under general anesthesia in a multicenter setting using the open technique. According to Novitsky's description [24], shown in Rosen's atlas of abdominal wall reconstruction [25], the non-viable Musculo-fascial tissue was debrided. Tension at the wound edges was assessed by applying the Kocher's clamps over the tissues, checking for sutures cutting through the tissues, and maintaining peak airway pressure readings below 12 mmHg [23]. After carefully separating the viscera from the wound edge (Additional file 2: Fig. S2), the posterior rectus sheath is located and incised around 1 cm from its edge, often at the umbilicus level (Additional file 3: Fig. S3). The retro-muscular plane is developed cranially by sharp dissection toward the xiphoid, caudally toward the pubis, and laterally to the linea semilunaris (Additional file 4: Fig. S4), preserving perforating neurovascular bundles that innervate and supply the rectus muscle (Additional file 5: Fig. S5). The dissection's extent depends on the wound's size and the actual dehiscence distance. The transverse abdominis muscle (TAM) is dissected from the peritoneum and the transversalis fascia by diathermy or harmonic scalpel (Additional file 6: Fig. S6) till reaching psoas major muscle (Additional file 7: Fig. S7). After performing both sides of release, the posterior rectus sheaths are re-approximated in the midline (Additional file 8: Fig. S8) with a continuous monofilament polydioxanone United States Pharmacopeia (USP) 1 on a TP-1 needle. Jenkins rule was used. We used Ethicon polyprolene mesh (30 × 30 cm, prolene brand, square with PMH code) inserted in the retro-muscular space and extending beyond the TAR. The mesh is secured using transfacial sutures with buried knots; additionally, the inferior edge of the mesh is secured to both cooper's ligaments via 2–4 interrupted monofilament sutures (Additional file 9: Fig. S9). The area is irrigated with vancomycin (2 g) and gentamycin 80 mg in 500 ml warm saline. Finally, after two suction drains are inserted on top of the mesh, the anterior rectus sheath is closed with a continuous monofilament polydioxanone USP 2–0 on an MH-1 needle (PDS II, Ethicon, Norderstedt, Germany) (Additional file 10: Fig. S10).

Post-surgery decisions about intensive care unit (ICU) admission are usually made by intensivists, surgeons, and anesthesiologists, and they are usually known by the end of surgery. The decision for ICU admission was according to the patient's general health before surgery, complications during surgery, the patient's age, obesity, the presence of multiple comorbidities, unstable cardiorespiratory function, long anesthesia time, preoperative ASA III, and intraoperative blood loss.

Postoperatively, patients were kept nil per oral and on intravenous fluids until bowel recovery.

Daily assessment for the following parameters (in addition to the vital signs): abdominal distention, drain output, AWD, SSI, seroma formation, and pus from one or more sites. In all patients, a postoperative abdominal binder was placed. The postoperative follow-up interval was one month, six months, one year, and every six months after that (The follow-up period was four years). Patients were assessed via email, phone, and outpatient clinic. Any complications were assessed by clinical examination and additional imaging studies and dealt with in due time. At one year, abdominal computed tomography (CT) scans were performed routinely to detect any occult IH, particularly in asymptomatic patients.

### Statistical analysis

SPSS 28 was used to manage and analyze data (IBM, Armonk, New York, United States). Quantitative data normality was determined using Kolmogorov–Smirnov, Shapiro–Wilk, and direct data visualization. Categorical data are presented with numbers and percentages. Quantitative and qualitative data were compared using the Chi-square and independent t-test, respectively. Multivariate logistic regression analysis determined the odds ratio and 95% confidence interval for incisional hernia predictors. All tests were two-sample. *P*-value < 0.05 was considered significant. The selection of variables in the model was made based on knowledge and clinical experience that produces a better model. We considered variables that are anticipated to cause an incisional hernia. Each variable was evaluated, controlling for the effect of possible and well-known confounders using ENTER method. Each variable was separately evaluated as we had a low incidence of incisional hernia (18 patients), not allowing to include many predictors in one model and may lead to a non-robust estimate. Additionally, to avoid multicollinearity, a very common, well-known problem in the case of multiple predictors. Multicollinearity can destroy a regression model and reverse the effect of predictors on the outcome.

## Results

**Fig. 2:**
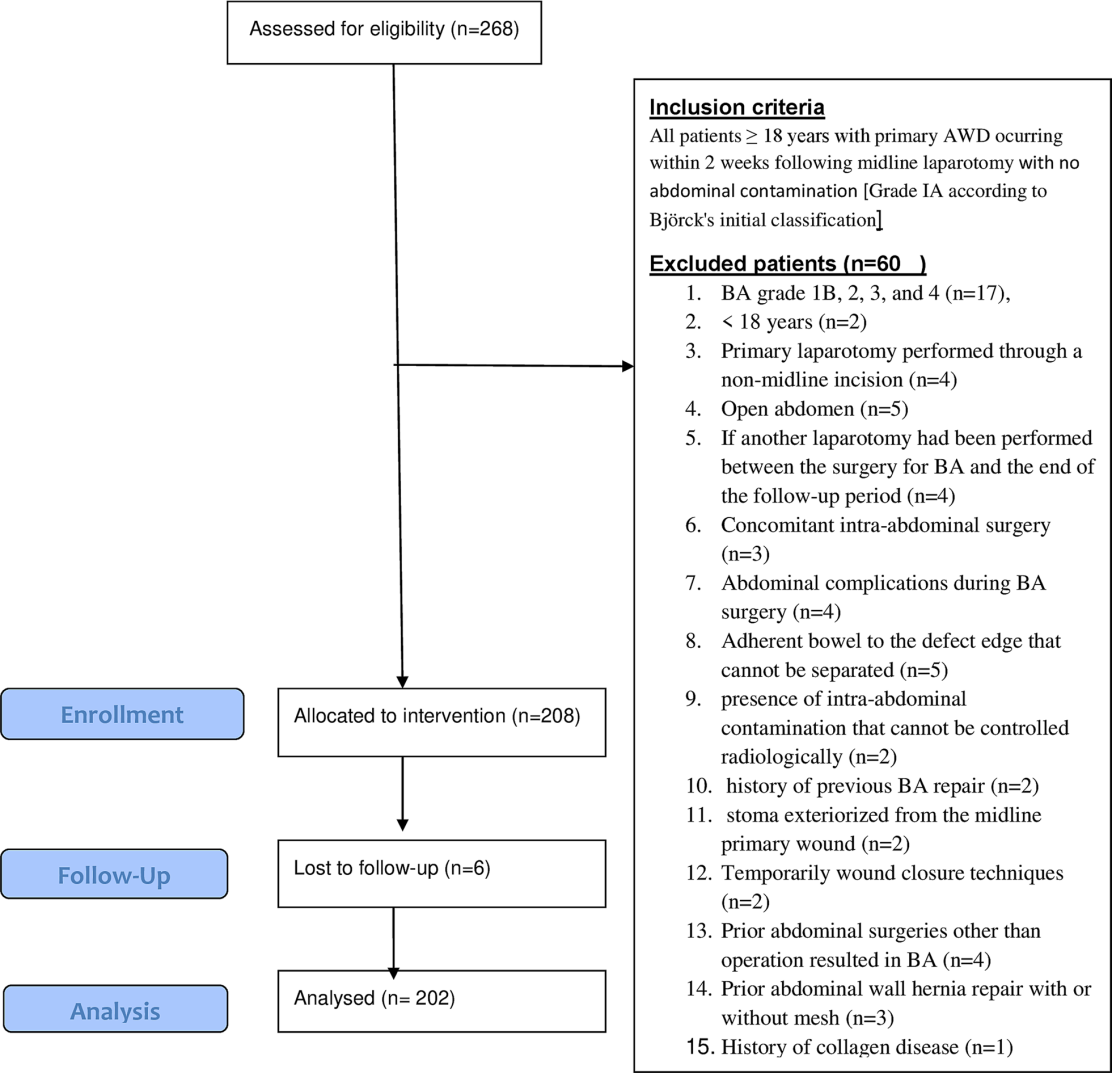
Flow diagram of inclusion and exclusion patients

A flow chart of inclusion and exclusion criteria was included (Fig. 2).

*Demographic and general characteristics of patients with primary AWD and candidates for posterior CS and TAR with retro-muscular mesh insertion*: The patients' mean age was 42 ± 10 years, with female predominance (59.9%). The mean body mass index (BMI) was 34 ± 4. Family history of hernia was reported in 15.3%. The mean time from index surgeries (midline laparotomies) till the occurrence of primary AWD was 7 ± 3 days. The most common site of the primary AWD was the umbilical (53%). About three-quarters of the patients had emergency surgery (72.3%). Cut-through sutures were the most frequent possible etiology of AWD (Table 2).

**Table 2:** Demographic and general characteristics of patients who developed burst abdomen and candidates for posterior Cs and TAR with retro muscular mesh insertion (202 patients)

General characteristics	*N* (%)
Age (years) (Mean ± SD)	42 ± 10
Gender	
Males	81 (40.1)
Females	121 (59.9)
Body mass index (Mean ± SD)	34 ± 4
ASA	
I	140 (69.3)
II	41 (20.3)
III	21 (10.4)
Diabetes mellitus	44 (21.8)
Hypertension	41 (20.3)
COPD	20 (9.9)
Smoking	46 (22.8)
Steroid intake	10 (5.0)
Family history of any previous hernia (weak mesenchyme)	31(15.3)
Time from index surgery(midline laparotomies) till primary abdominal wall dehiscence (days) (Mean ± SD)	7 ± 3
Albumin level < 3.5 gm %	46 (22.8)
Site of primary abdominal wall dehiscence (AWD)	
Infra umbilical	38 (18.8)
Supra umbilical	57 (28.2)
Umbilical	107 (53.0)
Previous operation resulting in primary AWD	
Emergent	146 (72.3)
Elective	56 (27.7)
Surgical speciality where primary AWD occurred	
General & GIT surgery	151 (74.8)
Gynecology	36 (17.8)
Vascular surgery	15 (7.4)
Possible etiology of burst	
Fascial necrosis	10 (5.0)
Infection	29 (14.4)
Loose knot	5 (2.5)
Cut through sutures	91 (45.0)
Unknown	67 (33.2)

*Intra-operative characteristics during posterior CS with TAR reinforced by retro-muscular mesh*: The median time from primary AWD to posterior CS with TAR surgery was 3 ± 1 days. AWD's median vertical length was 16 ± 2 cm. The mean operative time of posterior CS with TAR was 95 ± 12 min (Table 3).

**Table 3:** Intra-operative characteristics during posterior CS with TAR reinforced by retro muscular mesh (202 patients)

Operative characteristics	*N* (%)
Surgery Time from primary AWD to CS with TAR surgery (days) (Median ± SD)	3 ± 1
Operative time of primary AWD (minute) (Median ± SD)	95 ± 12
Patients with blood loss > 500 ml	41 (20.3)
Patients needed blood transfusion	42 (20.8)
Vertical length of primary AWD (cm) (Median ± SD)	16 ± 2
Horizontal length of primary AWD (cm) (Median ± SD)	12 ± 3
Hospital stay (days) (Median ± SD)	12 ± 1
Patients needed Intensive care unit admission	20 (9.9)

**Fig. 3:**
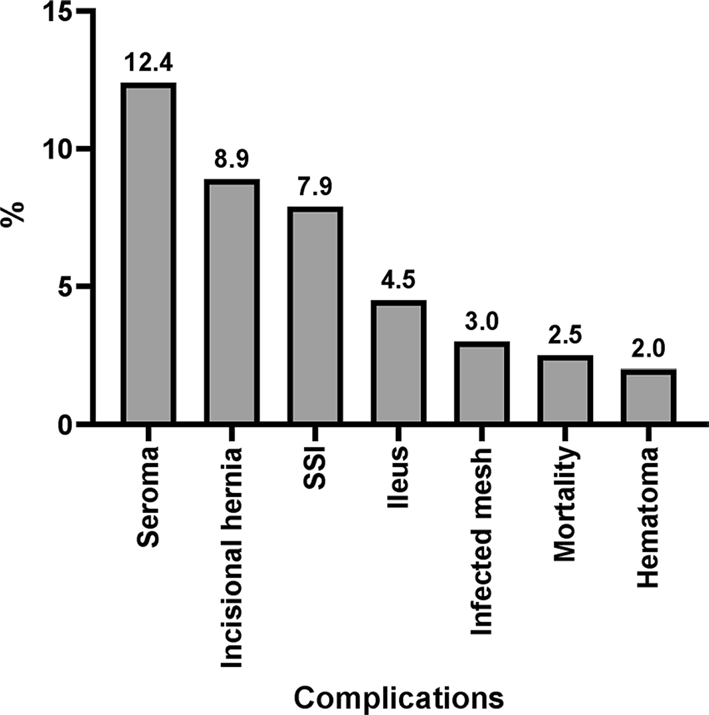
Complications in the studied patients

*Clavien-Dindo classification and complications after posterior CS with TAR reinforced by retro muscular mesh*: No complications were reported for 145 patients (72.8%). No cases of AWD recurrence. Only 7.9% of the patients had SSI, and 81.3% of them were superficial. Seroma was reported in 12.4% of the patients. The hematoma was reported in only 2%. The IH was reported in 8.9%, most of which were non-complicated (77.8%). Only 4.5% had ileus, and 3% had infected mesh. Mortality was reported in 2.5% (Table 4 and Fig. 3).

**Table 4:** Clavien-Dindo classification and postoperative complications following posterior CS with TAR reinforced by retro muscular mesh (202 patients)

	*N* (%)
Clavien-Dindo classification	
0 (No complications)	147 (72.8)
I	34 (16.8)
II	14 (6.9)
III	3 (1.5)
IV	4 (2.0)
Recurrent AWD	0 (00.0)
Surgical site infection (SSI)	
Type of SSI*	16 (7.9)
Deep	3 (18.8)
Superficial	13 (81.3)
Seroma	25 (12.4)
Hematoma	4 (2.0)
Incisional hernia following posterior CS + TAR	
Incisional hernia presentation**	18 (8.9)
Complicated hernia	4 (22.2)
Non complicated	14 (77.8)
Ileus	9 (4.5)
Infected mesh	6 (3)
Mortality	5 (2.5)

*Percentages calculated based on a total of 16 patients with SSI

**Percentages calculated based on a total of 18 patients with incisional hernia

*Risk factors for IH development after posterior CS with TAR reinforced by retro-muscular mesh*: The mean age (*P* < 0.001), male gender (*P* = 0.016), smoking (*P* < 0.001), albumin level < 3.5 gm % (*P* < 0.001), time from AWD occurrence to posterior CS with TAR surgery (*P* < 0.001), SSI (*P* < 0.001), ileus (*P* < 0.001) and infected mesh (*P* < 0.001) were significantly higher in those with an IH than without (Table 5).

**Table 5:** Risk factors for incisional hernia development after posterior CS with TAR reinforced by retro muscular mesh (*n* = 202)

	Incisional hernia		*P*-value
	Yes (*n* = 18) *N* (%)	No (*n* = 184) *N* (%)	
Age (years) (Mean ± SD)	55 ± 9	41 ± 9	< 0.001*
Gender			
Males	12 (66.7%)	69 (37.5%)	0.016*
Females	6 (33.3%)	115 (62.5%)	
Body mass index (Mean ± SD)	35 ± 3	34 ± 4	0.081
ASA			
I	10 (55.6%)	130 (70.7%)	
II	4 (22.2%)	37 (20.1%)	0.201
III	4 (22.2%)	17 (9.2%)	
Diabetes mellitus	6 (33.3%)	38 (20.7%)	0.213
Hypertension	6 (33.3%)	35 (19.0%)	0.15
COPD	3 (16.7%)	17 (9.2%)	0.314
Smoking	15 (83.3%)	31 (16.8%)	< 0.001*
Steroid intake	1 (5.6%)	9 (4.9%)	0.901
Family history of any previous hernia	1 (5.6%)	30 (16.3%)	0.227
Time from index surgeries (midline laparotomies) till occurrence of primary AWD (days) (Mean ± SD)	6 ± 2	7 ± 3	0.312
Albumin level < 3.5 gm%	16 (88.9%)	30 (16.3%)	< 0.001*
Site of AWD			
Infra umbilical	3 (16.7%)	35 (19.0%)	
Supra umbilical	8 (44.4%)	49 (26.6%)	0.268
Umbilical	7 (38.9%)	100 (54.3%)	
Previous midline laparotomies			
Emergent	13 (72.2%)	133 (72.3%)	0.996
Elective	5 (27.8%)	51 (27.7%)	
Specialty			
General & GIT	12 (66.7%)	139 (75.5%)	
Gynecology	4 (22.2%)	32 (17.4%)	0.76
Vascular	2 (11.1%)	13 (7.1%)	
Possible etiology of AWD			
Fascial necrosis	0 (0.0%)	10 (5.4%)	
Infection	3 (16.7%)	26 (14.1%)	
Loose knot	2 (11.1%)	3 (1.6%)	NA
Cut through suture	12 (66.7%)	79 (42.9%)	
Unknown	1 (5.6%)	66 (35.9%)	
Time from primary AWD to posterior CS + TAR surgery (days) (Mean ± SD)	5 ± 2	3 ± 1	< 0.001*
Operative time of posterior CS + TAR (minute) (Mean ± SD)	91 ± 10	95 ± 13	0.228
Blood loss > 500 ml	4 (22.2%)	37 (20.1%)	0.832
Blood transfusion (units)	4 (22.2%)	38 (20.7%)	0.876
Primary AWD vertical length (cm) (Mean ± SD)	16 ± 2	16 ± 3	0.746
Primary AWD horizontal length (cm) (Mean ± SD)	13 ± 3	12 ± 3	0.294
Hospital stay (days)(Mean ± SD)	12 ± 1	12 ± 1	0.682
ICU admission (days)	1 (5.6%)	19 (10.3%)	0.518
Surgical site infection	11 (61.1%)	5 (2.7%)	< 0.001*
Ileus	7 (38.9%)	2 (1.1%)	< 0.001*
Infected mesh	5 (27.8%)	1 (0.5%)	< 0.001*

Independent t-test was used for quantitative data. Chi-square or Fisher’s exact test was used for categorical data

*NA* Not applicable

*Significant difference

Multivariate logistic regression analysis controlling for age, gender, and BMI was done to predict IH. The model was built based on clinical experience. Predictors of IH were time from primary AWD occurrence till posterior CS with TAR surgical intervention (OR = 1.82, 95% CI = 1.25–2.65, *P* = 0.002), ileus (OR = 51.22, 95% CI = 6.33–414.59, *P* < 0.001), SSI (OR = 142.28, 95% CI = 16.78–1206.13, *P* < 0.001), and infected mesh (OR = 342.29, 95% CI = 16.45–7123.11, *P* < 0.001) (Table 6).

**Table 6:** Multivariate analysis for prediction of incisional hernia

	OR (95% CI)**	*P*-value
Steroid use	0.92 (0.08–9.04)	0.873
Diabetes mellitus	0.40 (0.11–1.48)	0.171
COPD	0.25 (0.05–1.28)	0.096
Time from primary AWD occurrence till posterior CS and TAR surgery (days)	1.82 (1.25–2.65)	0.002*
Ileus	51.22 (6.33–414.59)	< 0.001*
Surgical site infection after posterior CS + TAR surgery	142.28 (16.78–1206.13)	< 0.001*
Infected mesh (number)	342.29 (16.45–7123.11)	< 0.001*
Primary AWD vertical length (cm)	0.882 (0.695–1.119)	0.301
Primary AWD horizontal length (cm)	1.141 (0.937–1.390)	0.190
Operative time of posterior CS + TAR (minutes)	0.959 (0.901–1.02)	0.180
Emergent midline laparotomy surgery	11.223 (2.045–61.608)	0.005*

*OR* Odds ratio, *95% CI* 95% confidence interval

*Significant, **Adjusted for age, gender and BMI

**Fig. 4:**
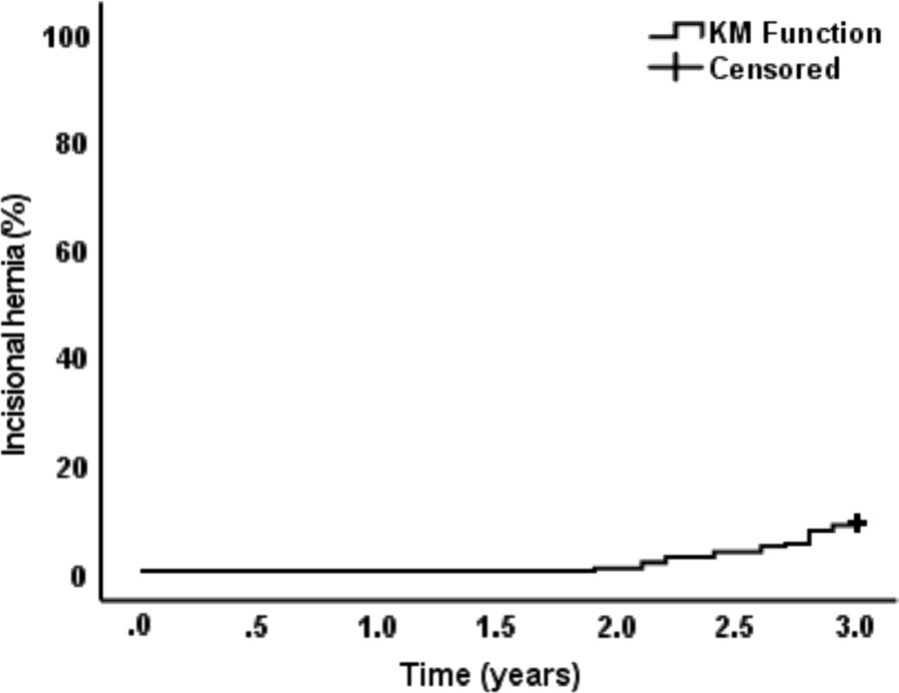
Kaplan Meier curve for time to incisional hernia

*Time to IH occurrence after posterior CS with TAR reinforced by retro-muscular mesh*: The Kaplan–Meier curve was used to estimate time to IH. At two years, the rate of IH was 0.5%. At three years, it was 8.9%. The median time to IH is shown in Fig. 4.

## Discussion

AWD is a serious surgical complication, but data regarding the best treatment is lacking, and the heterogeneity of the techniques makes comparisons between different therapies difficult [1, 14, 15]. Our research aimed to determine the efficacy of the posterior CS with TAR and retro-muscular mesh insertion in repair of primary AWD in terms of AWD recurrence, IH, SSO, and mortality. This is the first large-scale study of this technique's efficacy in treating AWD.

We reported no recurrence of AWD and an 8.9% incidence rate for IH at the three-year follow-up. The SSO risk for SSI, wound seroma, and wound hematoma was 7.9%, 12.4%, and 2%, respectively. A 2.5% mortality rate was observed. Cardiopulmonary complications were the causes of postoperative mortality (two patients with pulmonary embolism and three patients with myocardial infarction). Local wound complications did not cause the death. All the patients required long-term hospitalization after surgery. None required long-term intubation after repair. All of them died within three months of reconstructive surgery. Root cause analysis confirmed no preventable deaths.

Subramonia et al. evaluated vacuum-assisted wound closure (VAWCM) as a temporary wound cover. However, authors reported wound closure necessity in 39% of patients, logistical difficulties with repeated dressings every 2–3 days, enteric fistulae, IH (*n* = 12), prolonged hospital stay (39 days), and prolonged ICU stay (22 days) [5]. Heller et al. confirmed these disadvantages, except that few patients can be treated in outpatient clinics [6]. Other centers using VAWCM reported 70–100% successful fascial closure rates but high IH rates [7, 8]. Another study evaluated Bogota bag closure for AWD with similar disadvantages and a high mortality rate (28.6%) [26]. In our study, although posterior CS with TAR with mesh has the disadvantage of abdominal wall trauma and is a technically demanding procedure, closure of the AWD was successful in all cases and prevented exposure of the viscera, so there was no enterocutaneous fistula. A fistula can occur due to an iatrogenic injury to the bowel, but in our patients, careful adhesiolysis was performed. Also, the mesh is inserted in the retro-muscular space away from the abdominal viscera, thus reducing the risk of bowel erosion from contact with the mesh. In contrast to VAWCM, the posterior CS with TAR and retro-muscular mesh insertion has no AWD recurrence, a low incidence of IH (8.9%), a shorter hospital stay (12 ± 1 days), and is potentially less expensive. Multiple previous studies agreed with our results regarding the role of posterior CS with TAR as efficient methods for closure of AWD as regards low IH rates up to 8% but with 15% SSI. They attributed the low recurrence hernia rate due to the mesh inserted in retromuscular space [27–29].

Other studies evaluated mass closure with or without retention sutures [11, 12], elastic silicone U-shaped loop sutures [13], and a mass closure technique with 3 cm “large bites” in 5 mm “small steps” [10]. These techniques were not advocated due to the inconvenience, pain, and high prevalence of IH (25–43%) caused by ischemia at the defect edge by the loop, while the previous study [10] showed a recurrence of AWD in 13% of patients.

Our study did not use a retention suture as a supportive treatment. Instead, we addressed the retracted fascial defect by dissection of the preperitoneal space by TAR, and this enabled us to approximate both the anterior and posterior rectus sheath medially easily, even after the debridement of the ischemic fascial edge. Furthermore, our results showed that in 91 patients (45% of cases), the cause of AWD was cut through sutures due to the tension closure. Retraction of the fascial defect prevents easy closure of the fascial defect, so our main goal was to approximate the anterior and posterior fascial sheath without tension. Obtaining adequate durability of repair of AWD is one of the main goals in AWD surgery, nil AWD recurrence and a low incidence of IH in our series is comparable to the previous studies [10–12]. Other possible explanations for the low incidence of IH in our study include preserving perforating neurovascular bundles during dissection and using abdominal binders to support the wound in all cases during the postoperative period.

Previous research suggested that the cause of AWD could be the cutting of sutures through tissues [1, 30] or intra-abdominal abscesses [31], or impaired facial tissue quality [10, 14, 15]. Due to proper selection, no cases in our study demonstrated intra-abdominal abscesses. Additionally, fascial necrosis was reported in 5% of cases, and easy medialization of the fascial defect in our technique helps debridement of necrotic fascia without tension on closure.

The high recurrence and IH following AWD treatment may support mesh repair. We believe that mesh augmentation, when indicated, is an effective adjunct to AWD closure method, potentially lowering the risk of IH. Our series has confirmed the importance of mesh placement in the retro-muscular space because placing the mesh in this location helps mesh fixation to the posterior surface of the rectus muscle even when the intra-abdominal pressure is increased.

Paterson et al. stated that retro rectal mesh to close the AWD was associated with low IH but increased wound complications [4]. This concept was confirmed by Van’t et al. [32], while Scholtes et al. confirmed the opposite results, with a better outcome even in intra-abdominal infection [3].

EHS clinical guidelines recommended slowly absorbable continuous monofilament sutures following suture wound/wound length over four (i.e., PDS) for AWD closure with mesh augmentation whenever fascial closure is possible. They did not recommend a particular mesh or insertion site, but SSO may increase. CS must be chosen carefully. They also noted the lack of supporting data [17]. Our results suggest that posterior CS can safely and effectively manage AWD with TAR reinforced by retromuscular mesh with low morbidity and mortality.

In our series, SSI is low in incidence, probably due to the selection of cases of AWD (Grade 1A). Infected mesh occurred in 3% (6 patients) presented by sinuses discharging trivial pus, and all were cured with conservative management within three months of diagnosis. Cohort type may explain the low incidence of chronic SSI. Furthermore, our surgical technique included a sharp dissection of the retro-muscular space, suture ligation of blood vessels and harmonic scalpel rather than diathermy, and drain placement and removal only when the effluent volume was less than 20–50 cc, and finally, abdominal binder placement in all cases. Studies have confirmed the important role of the abdominal binder in preventing SSO and IH [33, 34]. However, other study denied this role [35]. We recommend that mesh be added to the posterior CS to reduce the incidence of IH and prevent AWD recurrence, even at the expense of SSO, which appears to be expected, but most SSO is self-limited.

Our study confirmed that time from AWD to surgery, emergency surgery, infected mesh, ileus, and SSI are predictors of IH as almost 74.8% of the AWD appeared after emergency laparotomy, which is higher than in previous studies (30–55%) [4, 36]. This could be explained by the fact that surgical strategies vary between centers. Our results confirmed that the length of the fascial defect and operative time were not risk factors for IH, probably due to adequate release of the anterior and posterior rectus sheath with tension-free closure of the fascial defect. These predictive factors are important to be considered by surgeons to minimize surgical repair failure.

### Strength and limitation

This study did not exclude emergency surgery or obese patients with the highest risk of AWD recurrence.

It is a consecutive series of patients, and selection bias was largely eliminated. The patients who lost to follow-up are also excluded, and this may skew the results as some of these patients might have developed complications and been treated elsewhere. Surgical experience is another factor that could have affected the outcomes, but all operations were conducted by consultant surgeons. The study does not compare various interventional techniques. Our future aim is to plan a study to compare the outcomes of our technique with other standard procedures.

This study did not assess any potential risks associated with future abdominal wall surgical therapy or the possibility of a negative effect on core abdominal wall and spine stability.

## Conclusion

This study adds the importance of obsessive attention to the sterility of the procedure, filling a step for evaluation of posterior CS + TAR surgery for repair of this emergent condition hoping to find most suitable approach and write shared guidelines in the surgical community. Posterior CS with TAR reinforced by retro-muscular mesh improves grade IA AWD outcomes. Long-term follow-up studies are needed to validate our results. Our technique resulted in no recurrent AWD and low IH rates comparable to others.

## Supplementary Information

**Additional file 1: Fig. S1.** Preoperative picture of burst abdomen.

**Additional file 2: Fig. S2.** Trimming of the edge of skin and fascia revealed retracted fascial edge with large defect.

**Additional file 3: Fig. S3.** Posterior component separation starts with division of posterior rectus sheath 1 cm from linea alba.

**Additional file 4: Fig. S4.** Dissection continues in retrorectal space till linea similunaris with preservation of neurovascular bundles supplying rectus muscle.

**Additional file 5: Fig. S5.** Close view of neurovascular bundles supplying rectus muscle.

**Additional file 6: Fig. S6.** Transversus abdominis muscle release by diathermy but may be by harmonic scalpel.

**Additional file 7: Fig. S7.** Dissection continued in periperitoneal space till psoas major muscle.

**Additional file 8: Fig. S8.** Approximation of posterior rectus sheath and sutured easily in midline with a continuous monofilament polydioxanone United States Pharmacopeia (USP) 1 on a TP-1 needle.

**Additional file 9: Fig. S9.** Solitary 30 × 30 cm polyprolene mesh is fixed in diamond pattern over closed posterior rectus sheath with 2 suction drains over it.

**Additional file 10: Fig. S10.** Closure of anterior rectus sheath over the mesh, with a continuous monofilament polydioxanone USP 2-0 on an MH-1 needle (PDS II, Ethicon, Norderstedt, Germany).

## Data Availability

Data are available on request.
