# Supplementary figures and images for: A prospective multicentre study evaluating the outcomes of the abdominal wall dehiscence repair using posterior component separation with transversus abdominis muscle release reinforced by a retro-muscular mesh: filling a step
Source: World J Emerg Surg. 2023 Mar 3;18:15. doi: 10.1186/s13017-023-00485-9 (PMC9985288; doi:10.1186/s13017-023-00485-9)

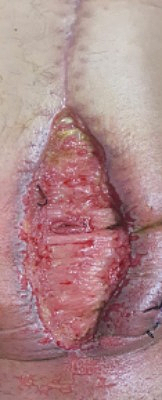

Supplement: Supplementary file 1 — Additional file 1: Fig. S1. Preoperative picture of burst abdomen. [file 13017_2023_485_MOESM1_ESM.tiff]

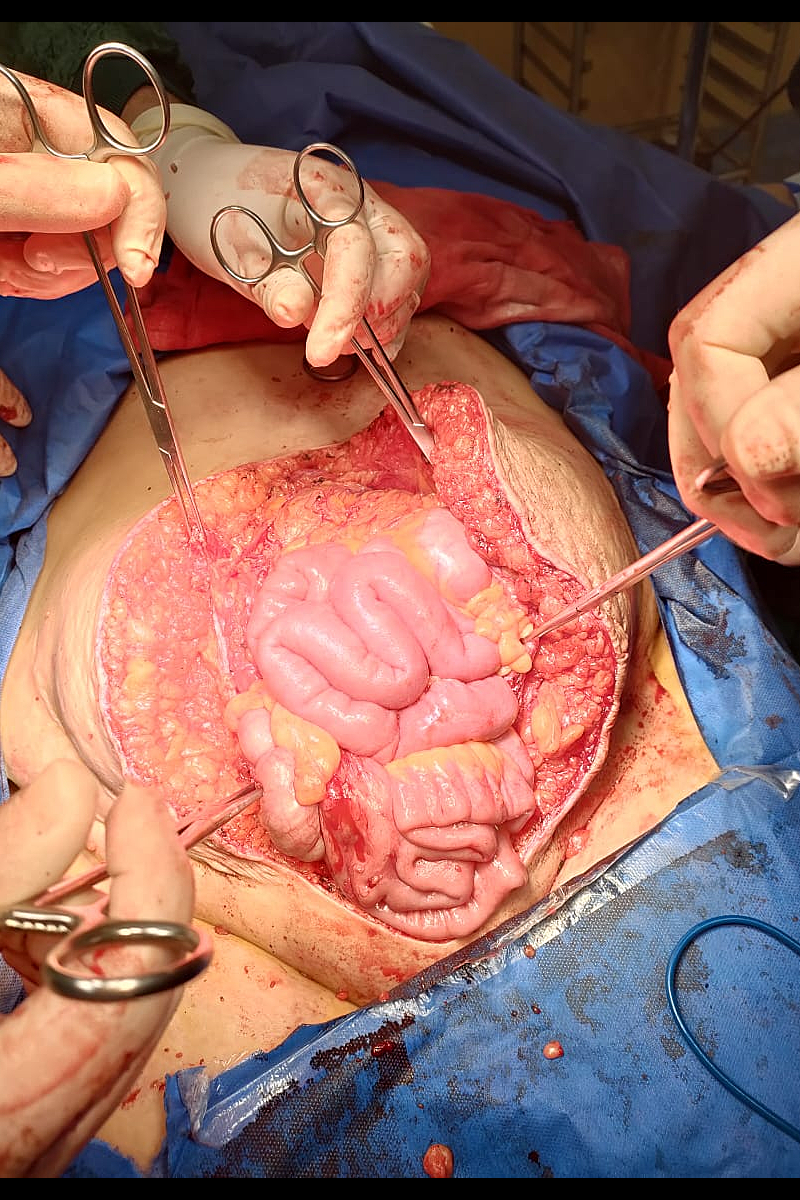

Supplement: Supplementary file 2 — Additional file 2: Fig. S2. Trimming of the edge of skin and fascia revealed retracted fascial edge with large defect. [file 13017_2023_485_MOESM2_ESM.tif]

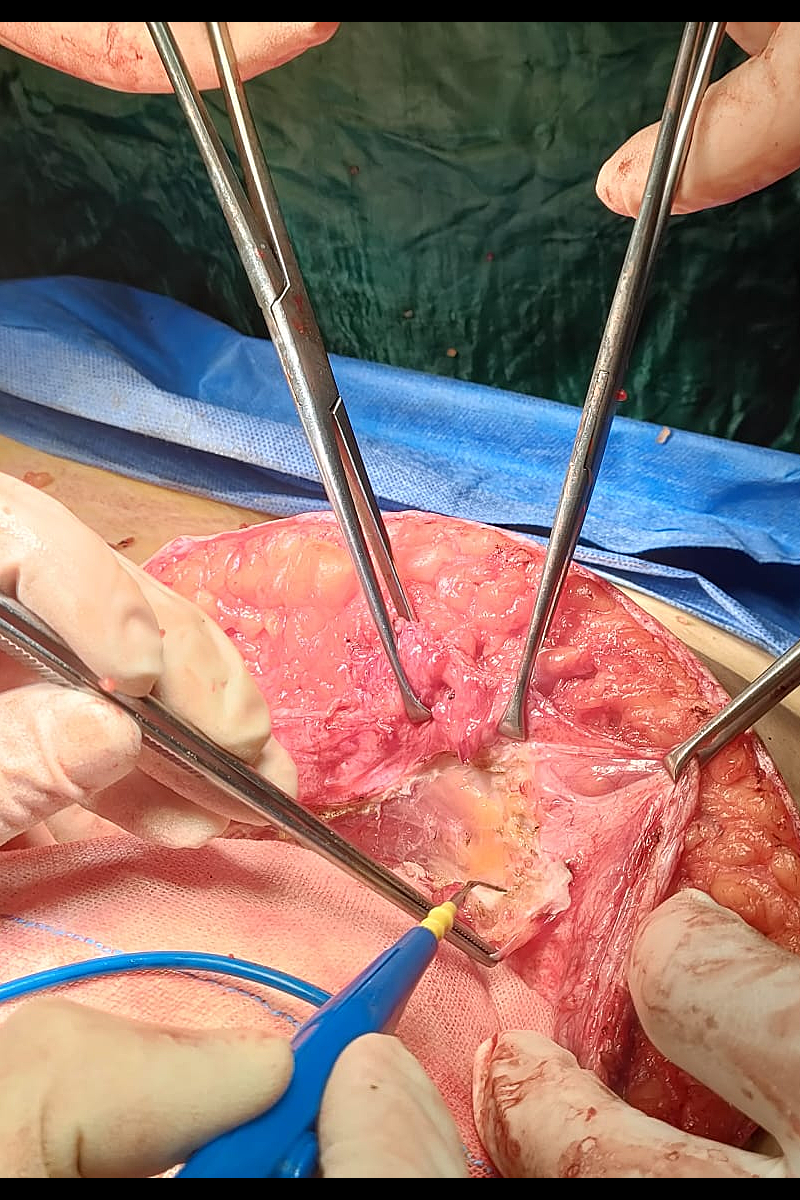

Supplement: Supplementary file 3 — Additional file 3: Fig. S3. Posterior component separation starts with division of posterior rectus sheath 1 cm from linea alba. [file 13017_2023_485_MOESM3_ESM.tif]

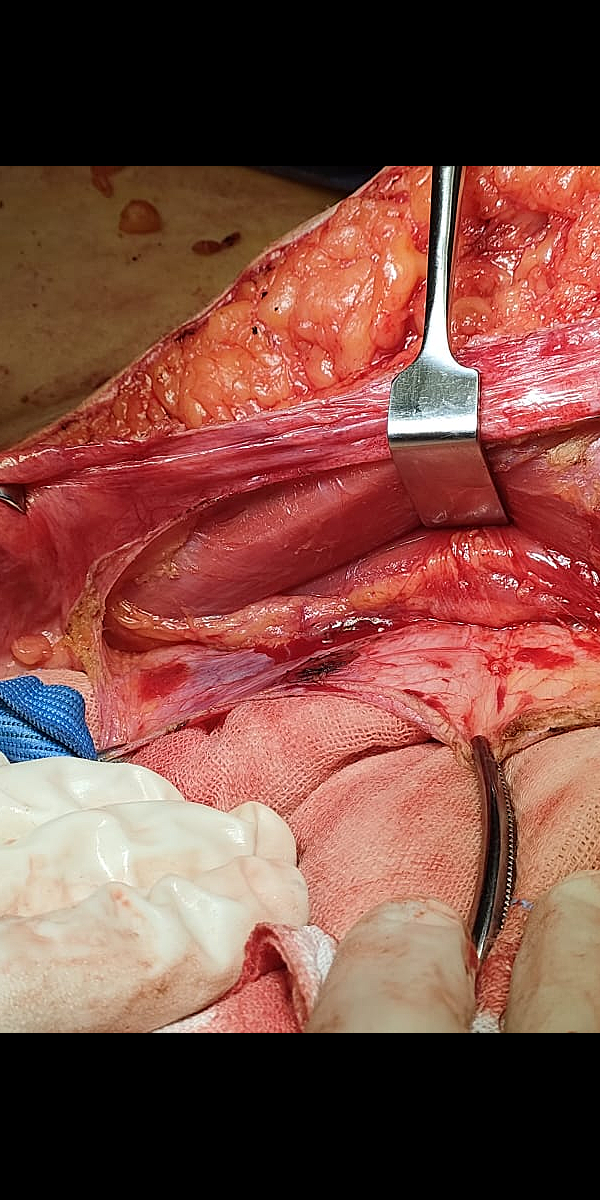

Supplement: Supplementary file 4 — Additional file 4: Fig. S4. Dissection continues in retrorectal space till linea similunaris with preservation of neurovascular bundles supplying rectus muscle. [file 13017_2023_485_MOESM4_ESM.tif]

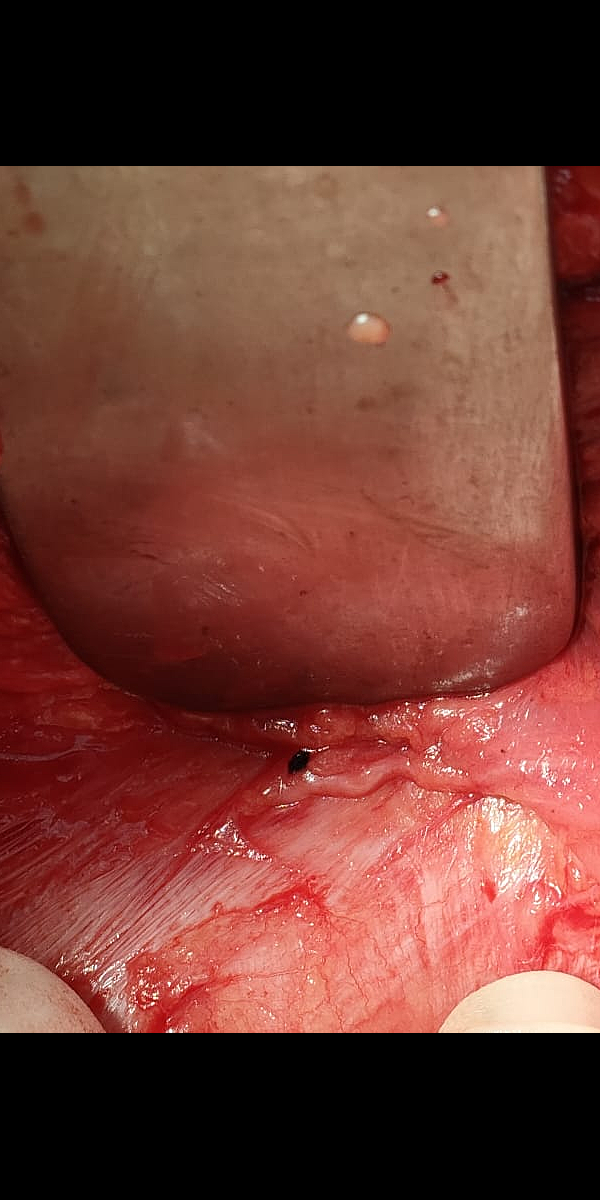

Supplement: Supplementary file 5 — Additional file 5: Fig. S5. Close view of neurovascular bundles supplying rectus muscle. [file 13017_2023_485_MOESM5_ESM.tif]

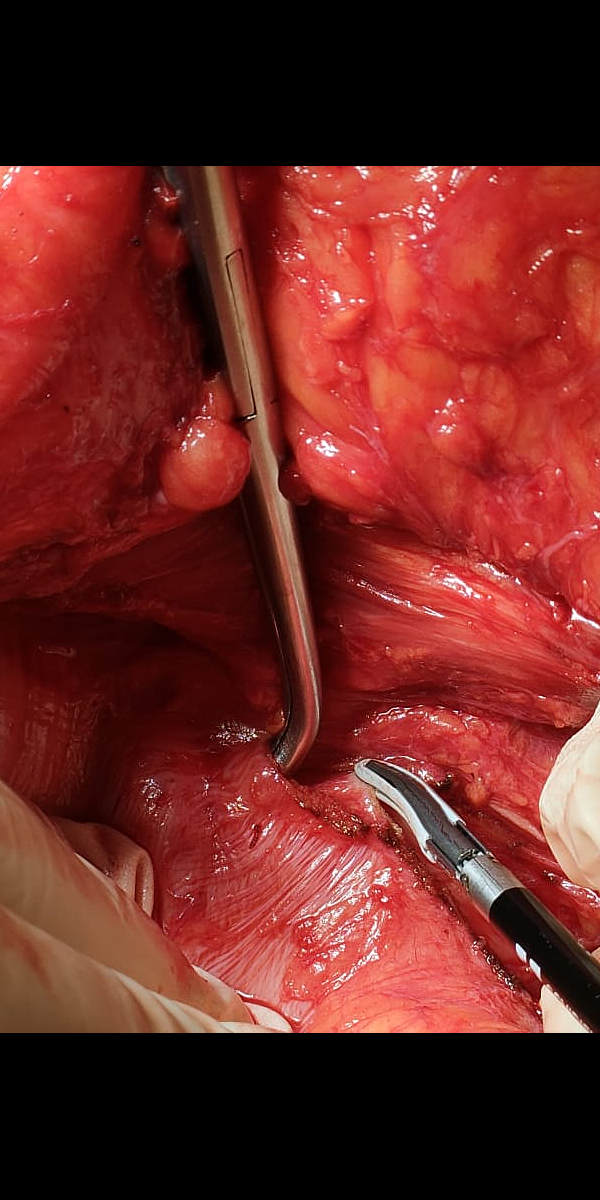

Supplement: Supplementary file 6 — Additional file 6: Fig. S6. Transversus abdominis muscle release by diathermy but may be by harmonic scalpel. [file 13017_2023_485_MOESM6_ESM.tif]

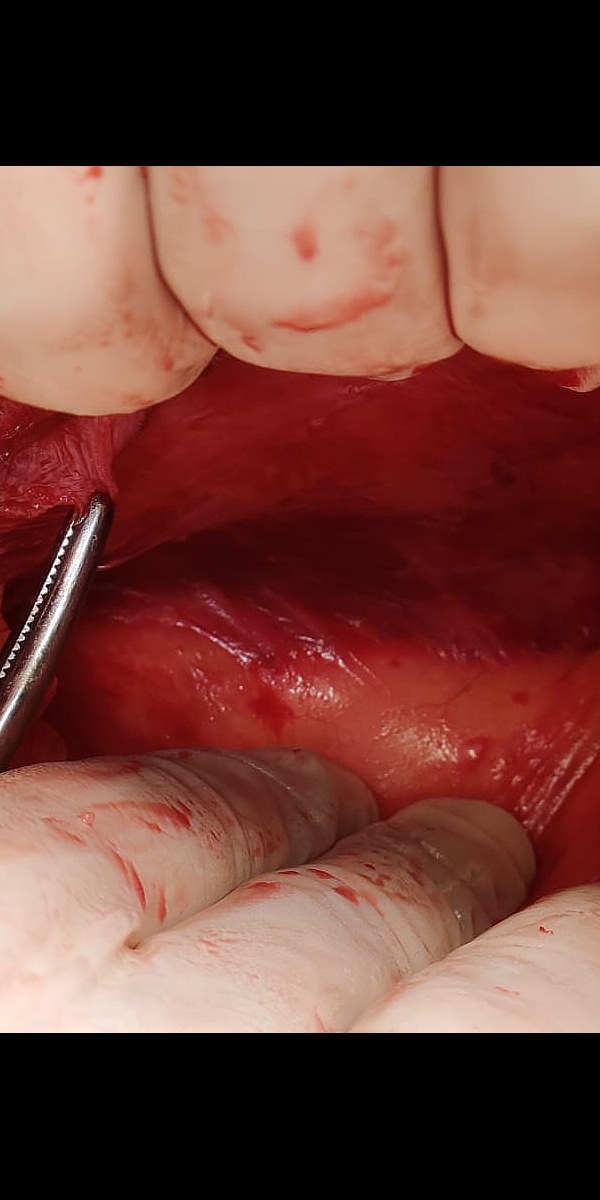

Supplement: Supplementary file 7 — Additional file 7: Fig. S7. Dissection continued in periperitoneal space till psoas major muscle. [file 13017_2023_485_MOESM7_ESM.tif]

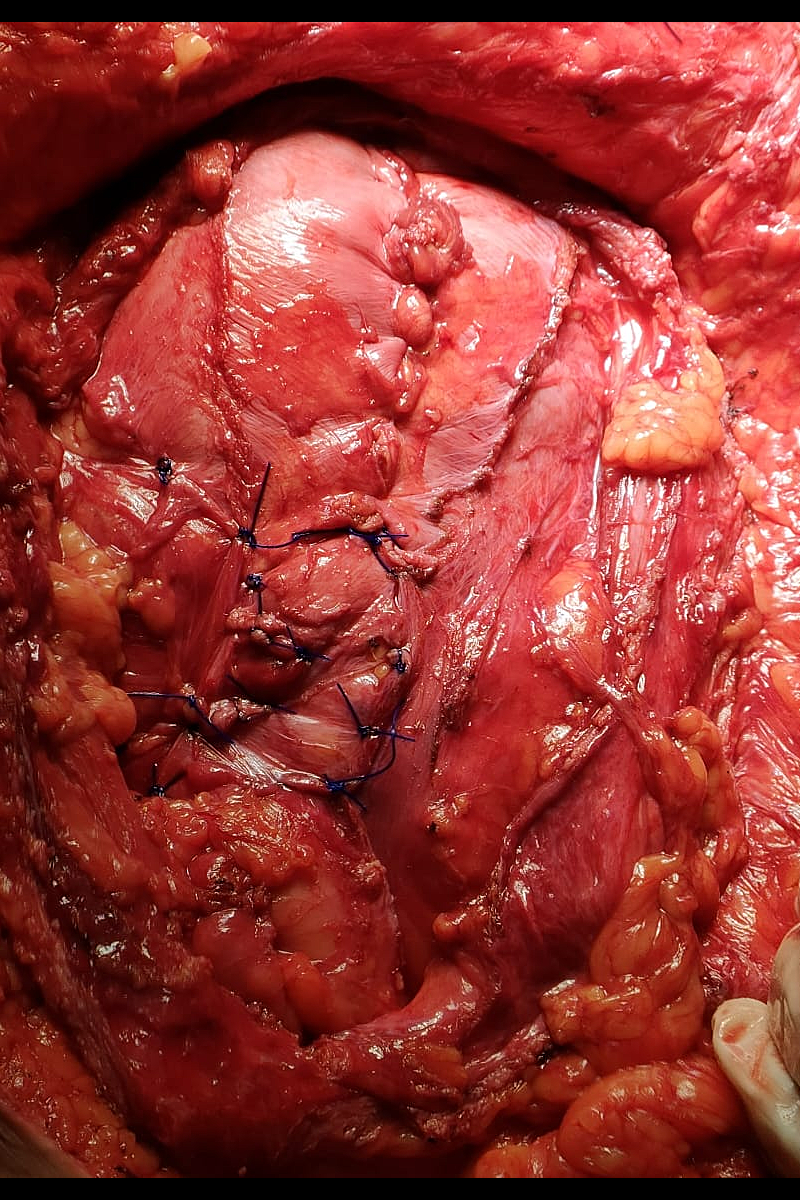

Supplement: Supplementary file 8 — Additional file 8: Fig. S8. Approximation of posterior rectus sheath and sutured easily in midline with a continuous monofilament polydioxanone United States Pharmacopeia (USP) 1 on a TP-1 needle. [file 13017_2023_485_MOESM8_ESM.tif]

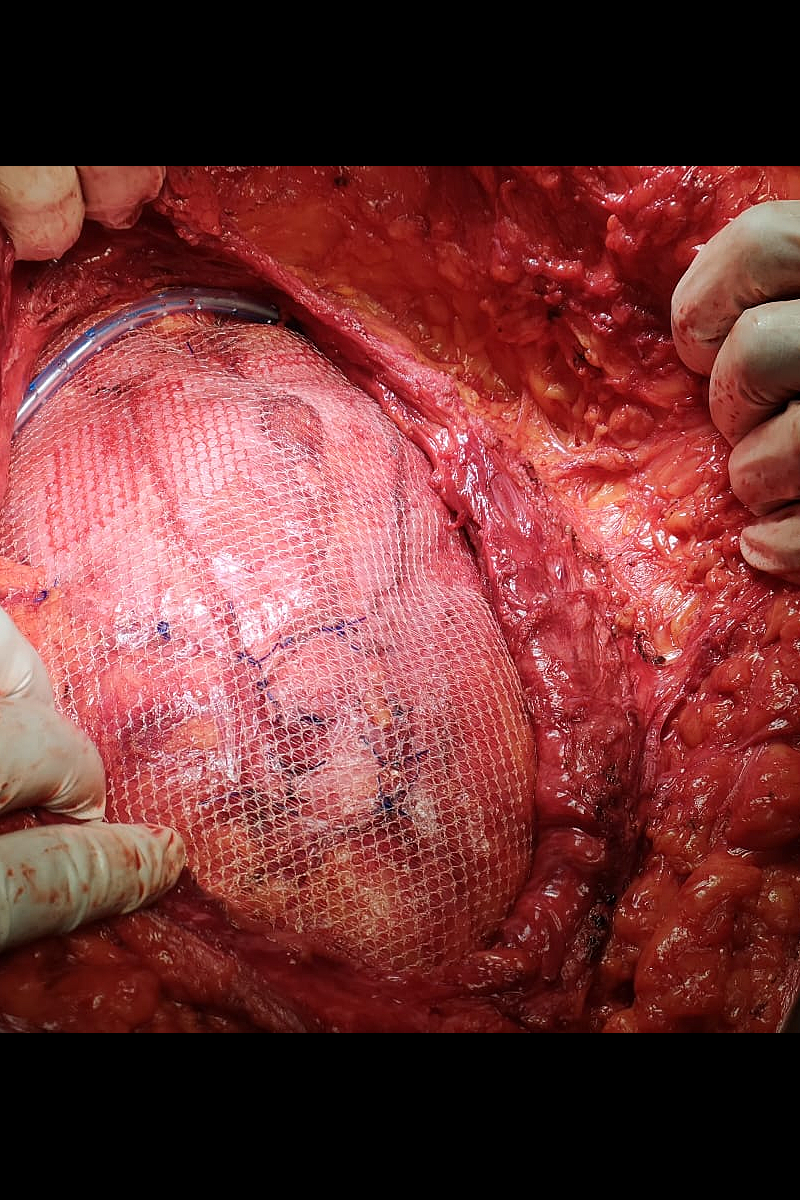

Supplement: Supplementary file 9 — Additional file 9: Fig. S9. Solitary 30 × 30 cm polyprolene mesh is fixed in diamond pattern over closed posterior rectus sheath with 2 suction drains over it. [file 13017_2023_485_MOESM9_ESM.tif]

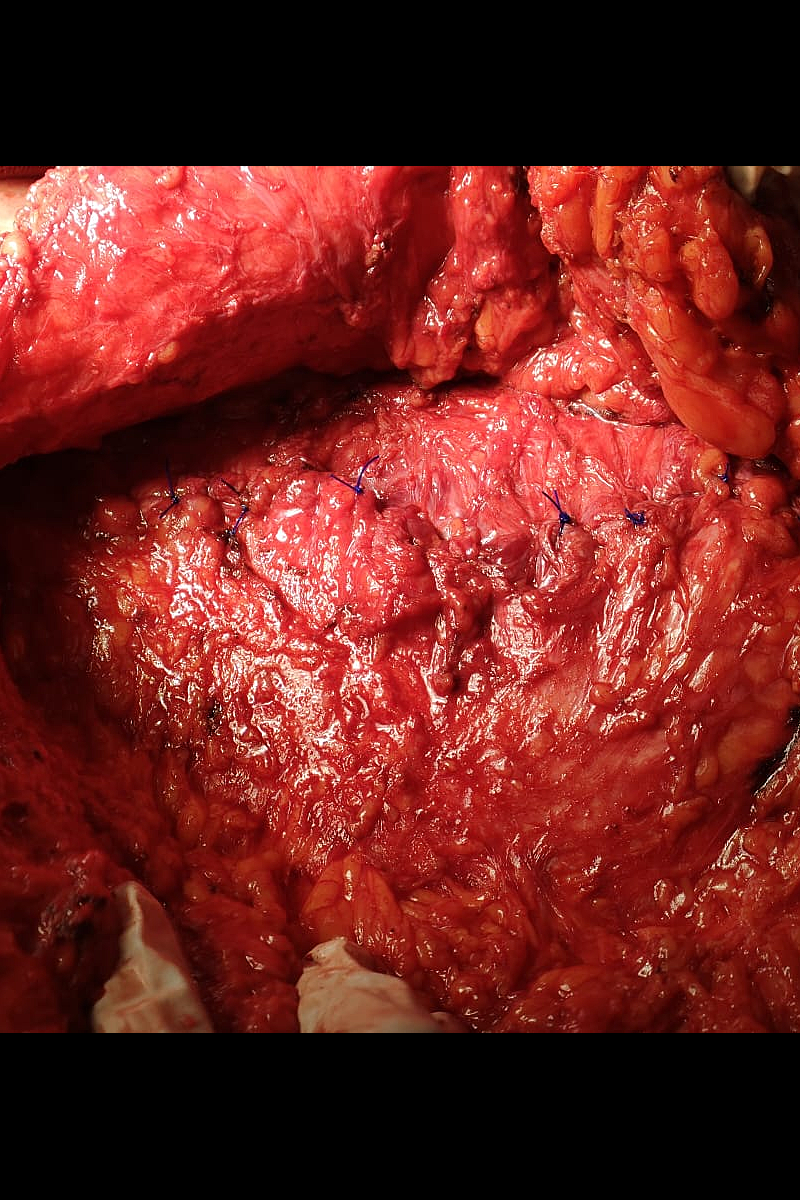

Supplement: Supplementary file 10 — Additional file 10: Fig. S10. Closure of anterior rectus sheath over the mesh, with a continuous monofilament polydioxanone USP 2-0 on an MH-1 needle (PDS II, Ethicon, Norderstedt, Germany). [file 13017_2023_485_MOESM10_ESM.tif]
